# Supplementary figures and images for: Hypertrophic Osteopathy Concurrent with an Aberrant Right Subclavian Artery in a Dog
Source: Vet Sci. 2024 Jun 7;11(6):263. doi: 10.3390/vetsci11060263 (PMC11209225; doi:10.3390/vetsci11060263)

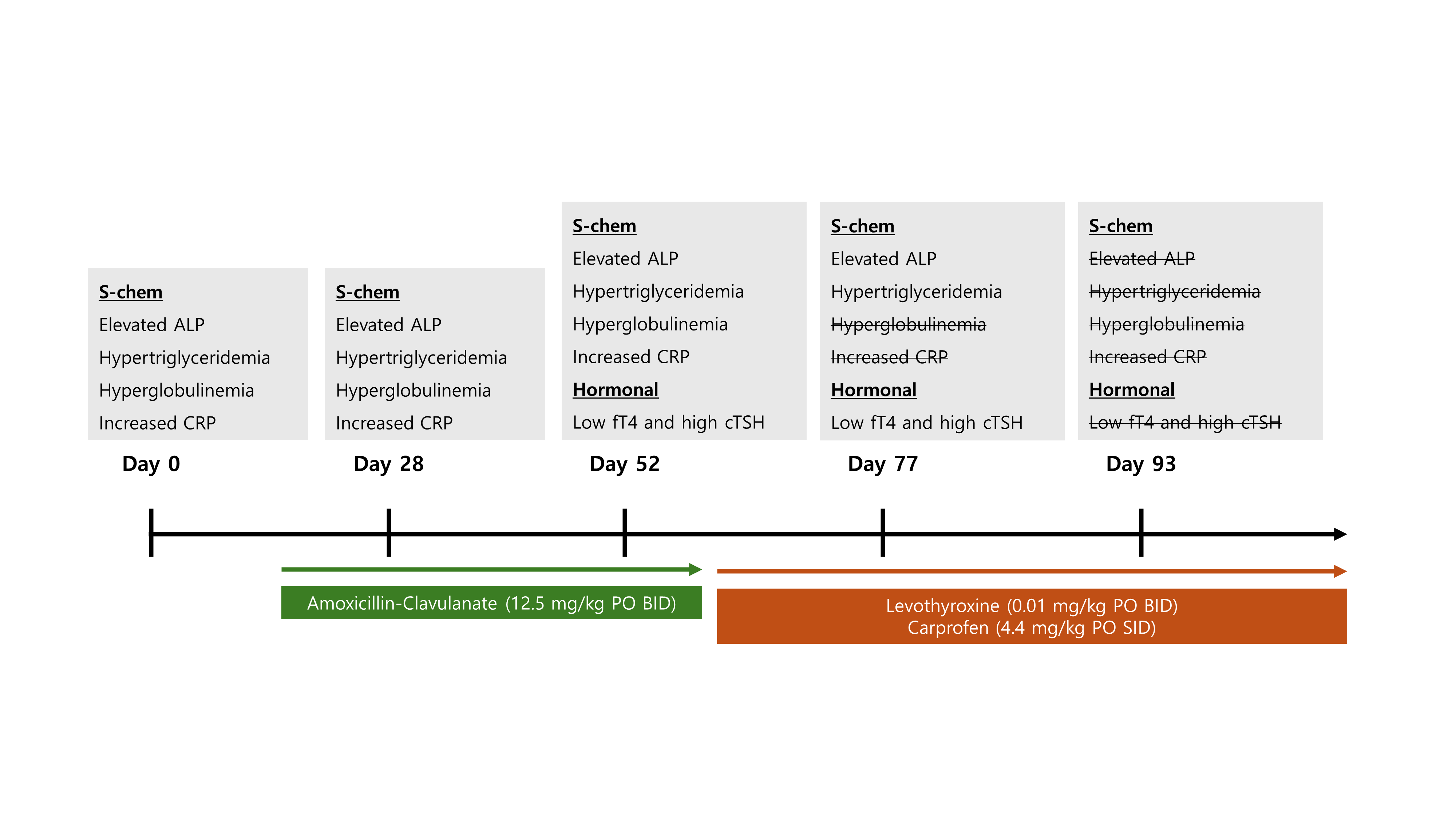

Supplement: Supplementary file 1 [file vetsci-11-00263-s001.zip › vetsci-3028595-supplementary.tif]
